# Nivolumab in Metastatic Non–Small Cell Lung Cancer

**Published:** 2016-03-01

**Authors:** Andrea Deel

**Affiliations:** Kingsport Hematology Oncology, Kingsport, Tennessee

Lung cancer is the leading cause of cancer deaths in the United States (American Cancer Society [ACS], 2015). Non–small cell lung cancer (NSCLC) is one of the most common types of the disease and accounts for 85% to 90% of lung cancer cases (ACS, 2015). Nonsquamous NSCLC accounts for approximately 45% to 60% of all lung cancer cases, whereas squamous NSCLC accounts for approximately 25% to 30% of all lung cancer cases (ACS, 2015).

Survival rates vary depending on the stage and type of the cancer and when it is diagnosed. For stage IV NSCLC, the 5-year survival rate is 1% (ACS, 2015). Factors that influence treatments for an individual patient include histology (squamous vs. nonsquamous), age, comorbidity, performance status, and preferences and concerns regarding treatment. The presence or absence of driver mutations can be another factor. Patients should have tumor tissue assessed for the presence of a driver mutation, such as mutated epidermal growth factor receptor (EGFR) or the anaplastic lymphoma kinase (ALK) fusion oncogene (Kerr et al., 2014).

Ongoing research into the molecular pathways that drive malignancy in NSCLC has led to the development of agents that target specific molecular pathways in cancer cells (Kerr et al., 2014). Targeted therapies are a significant step forward in treating patients with tumors that contain specific mutations in these pathways.

In the United States, several agents have been approved to target these tumors. They include erlotinib, afatinib (Gilotrif), and gefitinib (Iressa), which target EGFR signaling; crizotinib (Xalkori) and ceritinib (Zykadia), which target *ALK* rearrangements; bevacizumab (Avastin) and ramucirumab (Cyramza), which target vascular endothelial growth factor (VEGF) signaling. Pembrolizumab (Keytruda) and nivolumab (Opdivo) target programmed cell death protein 1 (PD-1) signaling (Lexi-Comp, 2015). Nivolumab, one of the agents that target PD-1 signaling, has become of particular interest for its clinical activity against malignant cells.

The PD-1 protein is an immune checkpoint receptor expressed by activated T cells (Topalian et al., 2012; Brahmar et al., 2012). In a normal physiologic state, this immune checkpoint exists to protect against inflammation and autoimmunity (Robert et al., 2015). In a neoplastic state, PD-1 binds to its ligands PD-L1 (B7-H1) and PD-L2 (B7-DC), which are expressed on tumor cells; this inhibits T-cell proliferation, cytokine production, and cytolytic function, thereby causing immunosuppression and preventing the immune system from rejecting the tumor (Brahmer, Horn, & Antonia, 2013; Brahmer & Pardoll, 2013; Sundar, Soong, Cho, Brahmer, & Soo, 2014).

The overexpression of PD-L1 on CD8-positive T cells has been identified in NSCLC tumors and is associated with a poor prognosis (Hirahara et al., 2012; Konishi et al., 2004; Mu, Huang, Chen, Chen, & Zhang, 2011). Monoclonal antibodies, including nivolumab, target the binding of the PD-1 receptor to one of its ligands, which disrupts the negative PD-1–receptor signaling and augments the T-cell response (Robert et al., 2015).

## PHARMACOLOGY

Nivolumab is a fully human immunoglobulin G4 (IgG4) monoclonal antibody that selectively inhibits PD-1 activity by binding to the PD-1 receptor to block PD-L1 and PD-L2 from binding (Bristol-Myers Squibb, 2016). The negative PD-1–receptor signaling that regulates T-cell activation and proliferation is therefore disrupted (Robert et al., 2015). This releases PD-1 pathway–mediated inhibition of the immune response, including the antitumor immune response (Bristol-Myers Squibb, 2016).

## INDICATION AND DOSING

Nivolumab is approved in the United States for the treatment of unresectable or metastatic melanoma as a single agent in patients with disease progression following ipilimumab (Yervoy) and, if positive for *BRAF* V600 mutation, a BRAF inhibitor. It is also used in combination with ipilimumab in patients with *BRAF* V600 wild-type unresectable or metastatic melanoma (Postow et al., 2015; Larkin et al., 2015). Nivolumab is indicated for treatment of advanced metastatic renal cell carcinoma in patients who have received prior therapy (Bristol-Myers Squibb, 2016). Clinical trials have shown the efficacy of nivolumab in treating patients with NSCLC, which lead to its approval for this disease by the US Food and Drug Administration (FDA) in 2015 (Bristol-Myers Squibb, 2016).

The indication includes the treatment of metastatic NSCLC, for both advanced squamous NSCLC and nonsquamous NSCLC, that has progressed on or after platinum-based chemotherapy (Bristol-Myers Squibb, 2016). Patients who have genomic tumor aberrations, such as *EGFR* or *ALK*, and are receiving an approved EGFR- or ALK-directed therapy should have disease progression prior to receiving nivolumab (Bristol-Myers Squibb, 2016; Rizvi et al., 2015).

Adult dosing is 3 mg/kg once every 2 weeks as an infusion, mixed in 0.9% sodium chloride or 5% dextrose in water, to a final concentration of between 1 and 10 mg/mL. It is infused over 60 minutes using a low protein binding 0.2- to 5-µ inline filter (Bristol-Myers Squibb, 2016).

## CLINICAL TRIALS

Multiple clinical trials have been conducted to examine the efficacy of nivolumab in patients with advanced-stage NSCLC. CheckMate 017 is a phase III, open-label, randomized clinical trial that evaluated nivolumab and docetaxel. Patients with advanced squamous cell NSCLC who had progressed during or after a prior platinum-based chemotherapy regimen were randomized to receive either nivolumab at 3 mg/kg intravenously (IV) over 60 minutes every 2 weeks vs. docetaxel at 75 mg/m² IV administered every 3 weeks. The trial included patients regardless of their PD-L1 expression status (Bristol-Myers Squibb, 2016). The primary endpoint was overall survival (OS), and secondary endpoints included progression-free survival (PFS) and objective response rate (ORR; Brahmer et al., 2015; Bristol-Myers Squibb, 2016).

The results of the trial showed an OS benefit, with an estimated 28% of patients alive at 18 months for nivolumab vs. 13% for docetaxel. The median OS for the nivolumab arm was 9.2 months and 6.0 months for docetaxel (hazard ratio [HR]: 0.62; 95% confidence interval [CI] = 0.48–0.81; *p* = .0004; Brahmer et al., 2015; Bristol-Myers Squibb, 2016).

The PFS rate at 18 months was 17% for the nivolumab arm vs. 2.7% for docetaxel. Median PFS was 3.5 months for patients administered nivolumab vs. 2.8 months for docetaxel (HR: 0.63; 95% CI = 0.48–0.83; *p* = .0008). The ORR was 20% for the nivolumab arm vs. 9% for docetaxel, for an estimated odds ratio of 2.6 (95% CI = 1.3–5.5; *p* = .0083), with a continued ongoing response seen in 63% of patients treated with nivolumab (Brahmer et al., 2015; Bristol-Myers Squibb, 2016).

Adverse effects occurred less frequently with nivolumab (131 patients) with any grade, 59%, and grade 3 or more, 8%. In 129 patients treated with docetaxel, adverse effects for any grade were 87% and for grade 3 or greater, 58% (Brahmer et al., 2015; Bristol-Myers Squibb, 2016). These results included both hematologic and nonhematologic toxicities (Bristol-Myers Squibb, 2016).

Another clinical trial, CheckMate 057, a phase III, open-label, randomized clinical trial that evaluated patients with metastatic nonsquamous NSCLC who had experienced disease progression during or after one prior platinum-based chemotherapy regimen. This study included patients regardless of their PD-L1 expression status. Patients were randomized to receive either nivolumab at 3 mg/kg administered IV every 2 weeks or docetaxel at 75 mg/m² administered IV every 3 weeks (Borghaei et al., 2015; Bristol-Myers Squibb, 2016).

The primary endpoint of this trial was overall survival, which was 12.2 months in the nivolu-mab arm (95% CI = 9.7–15.0) and 9.4 months in the docetaxel arm (95% CI = 8.0–10.7). The HR resulted with 0.73 (95% CI = 0.60–0.89; *p* = .0015; Borghaei et al., 2015; Bristol-Myers Squibb, 2016). This translates to a 27% reduction in the risk of death with nivolumab vs. docetaxel (Bristol-Myers Squibb, 2016).

The ORR in the nivolumab arm was 19%, showing that 56 of 292 patients had responses: 4 complete responses and 52 partial responses (95% CI = 15–24; Borghaei et al., 2015; Bristol-Myers Squibb, 2016). Docetaxel had an ORR of 12%, demonstrating that 36 of 290 patients had responses—1 complete response and 35 partial responses (95% CI = 9–17; *p* = .02; Borghaei et al., 2015; Bristol-Myers Squibb, 2016). The median duration of response was 17 months in the nivolumab arm and 6 months in the docetaxel arm. Median PFS was 2.3 months in the nivolu-mab arm vs. 4.2 months with docetaxel (HR = 0.92; 95% CI = 0.77–1.11, *p* = .39; Borghaei et al., 2015; Bristol-Myers Squibb, 2016).

## ADVERSE EVENTS AND TOXICITY MANAGEMENT

In clinical trials, the most common adverse reactions (≥ 20%) reported by patients treated with nivolumab were fatigue (50%), dyspnea (38%), musculoskeletal pain (36%), decreased appetite (35%), cough (32%), nausea (29%), constipation (24%), and rash (21%; Bristol-Myers Squibb, 2016). Other adverse events may include edema; chest pain; and electrolyte imbalances including hyponatremia, hypokalemia, hyperkalemia, hyperglycemia, hypomagnesemia, hypocalcemia, and hypercalcemia (Lexi-Comp, 2015).

Severe infusion reactions have been reported in < 1% of patients in clinical trials of nivolumab as a single agent. Infusions should be interrupted or the rate of infusion slowed in patients with grade 1 or 2 reactions and discontinued in patients with grade 3 or 4 infusion reactions (Table 1). Immune-mediated adverse events included pneumonitis, colitis, rash, adrenal insufficiency, hypophysitis, thyroid dysfunction, encephalitis, and hepatitis (Bristol-Myers Squibb, 2016).

**Table 1:**
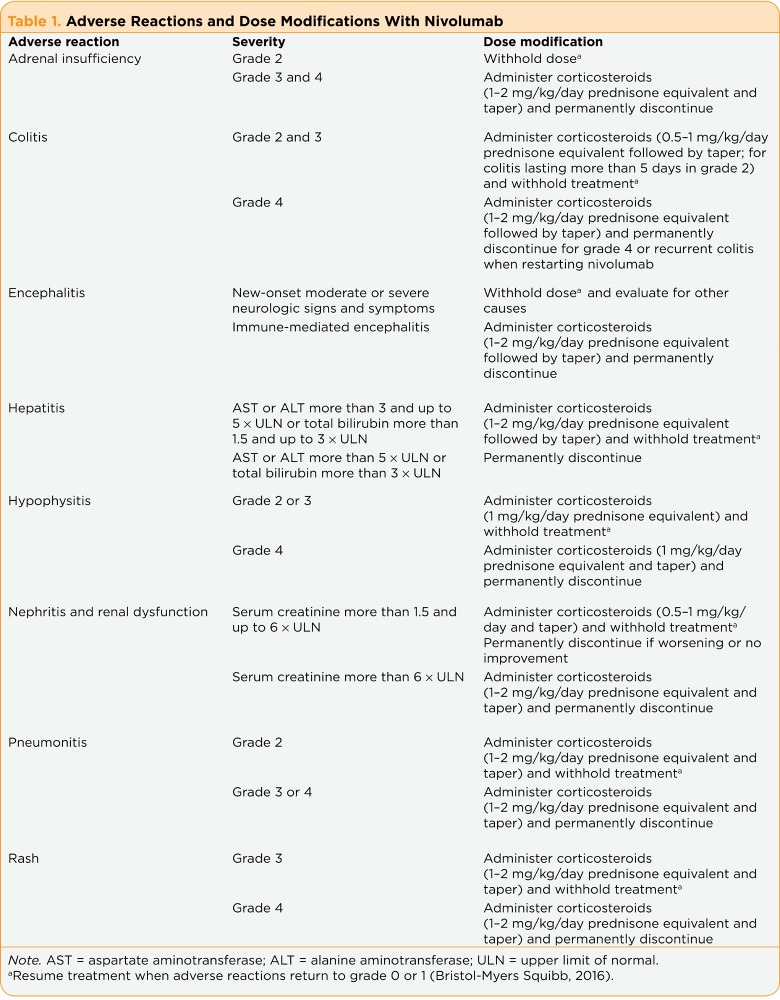
Adverse Reactions and Dose Modifications With Nivolumab

Baseline and periodic hepatic and renal function tests should be monitored (Lexi-Comp, 2015). Serum blood glucose levels should be assessed prior to each treatment and throughout therapy for patients at risk for hyperglycemia and patients with diabetes. Monitoring for signs and symptoms of hypophysitis and adrenal insufficiency during and after treatment is also indicated (Bristol-Myers Squibb, 2016).

Both hyperthyroidism and hypothyroidism should also be monitored prior to and every 6 weeks throughout therapy while treating accordingly. There are no dose adjustments with thyroid dysfunction (Bristol-Myers Squibb, 2016). In CheckMate 057, which included 287 patients, grade 1 or 2 hypothyroidism, which includes thyroiditis, occurred in 7% of patients, and elevated thyroid-stimulating hormone occurred in 17% of patients. Grade 1 or 2 hyperthyroidism occurred in 1.4% of patients receiving nivolumab (Bristol-Myers Squibb, 2016). Dose reductions and adjustments are indicated for other immune-mediated adverse events.

In clinical trials, fatal immune-mediated pneumonitis, or interstitial lung disease, occurred in 0.5% of 978 patients, and adrenal insufficiency occurred in 1% of a group of 555 patients who received nivolumab as a single agent. Other adverse events monitored in CheckMate 057 (n = 287) were diarrhea or colitis, occurring in 17% of patients receiving nivolumab. Immune-mediated colitis occurred in 2.4% of patients, including three grade 3 events. Grade 2 immune-mediated renal dysfunction occurred in one patient. Fatal limbic encephalitis also occurred in one patient. Immune-mediated rash occurred in 6% of patients receiving nivolumab, including four grade 3 cases (Bristol-Myers Squibb, 2016). One drug interaction is listed, stating concomitant use with belimumab is contraindicated due to enhancing adverse effects of belimumab (Lexi-Comp 2015).

## IMPLICATIONS FOR ADVANCED PRACTITIONERS

Nivolumab is currently approved by the FDA for treatment of metastatic NSCLC, for both advanced squamous NSCLC and nonsquamous NSCLC, that has progressed on or after platinum-based chemotherapy. Routine laboratory monitoring of patients receiving nivolumab should include hepatic and renal function tests, thyroid-stimulating hormone, and blood glucose. Monitoring for signs and symptoms of adrenal insufficiency, hypophysitis, thyroid disorders, diabetes, immune-mediated colitis, pneumonitis, rash, encephalitis, and infusion reactions should be done with each treatment and throughout therapy (Bristol-Myers Squibb, 2016).

Patients should be educated to tell their health-care provider if they have immune system problems such as Crohn’s disease, ulcerative colitis, or lupus; or have had lung or breathing problems, an organ transplant, liver problems, or any other medical condition. Patients should be informed that the most common side effects in patients with NSCLC treated with nivolumab are tiredness; pain in muscles, bones, and joints; decreased appetite; cough; and constipation. It is crucial to inform the patient to report to the health-care staff if symptoms of chills, shaking, itching, rash, flushing, difficulty breathing, dizziness, fever, and feeling of passing out during infusion of nivolumab. Patients should be monitored throughout therapy for adverse reactions, including immune-mediated adverse reactions (Table 2), and should notify the health-care provider if any symptoms develop (Bristol-Myers Squibb, 2016).

**Table 2:**
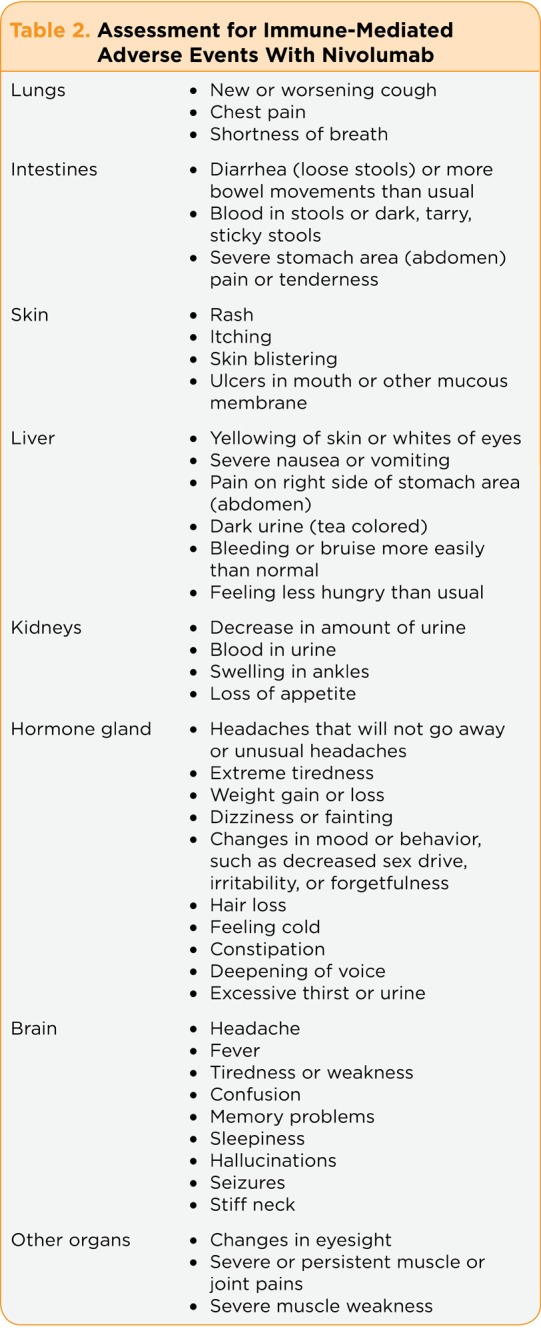
Assessment for Immune-Mediated Adverse Events With Nivolumab

## CONCLUSION

Research into the PD-1 pathway has been shown to be valuable in the immune system’s ability to control cancer. Blocking this pathway with targeted therapies such as nivolumab has led to tumor response and control in some patients with NSCLC. Nivolumab has been shown to be efficacious in clinical trials, with increases in overall and progression-free survival compared with docetaxel. The recent approval of nivolumab may give another option to patients with previously treated metastatic NSCLC who have progressed on prior treatments.

Trial results show nivolumab to be fairly well tolerated; however, due to the potential for serious adverse effects, patients receiving nivolumab should be monitored closely. Patients and health-care providers should be educated on nivolumab, including treatment regimen, monitoring parameters, adverse events, as well as immune-mediated adverse events, which may require dose modifications and treatment with corticosteroids.
